# 4,4-Dimethyl-2-tosyl-1,2,3,3a,4,11b-hexa­hydro-11*H*-pyrrolo[3,4-*c*]pyrano[5,6-*c*]chromen-11-one 0.125-hydrate

**DOI:** 10.1107/S1600536809044730

**Published:** 2009-10-31

**Authors:** K. Chinnakali, D. Sudha, M. Jayagobi, R. Raghunathan, Hoong-Kun Fun

**Affiliations:** a Department of Physics, Anna University Chennai, Chennai 600 025, India; b Department of Organic Chemistry, University of Madras, Guindy Campus, Chennai 600 025, India; c X-ray Crystallography Unit, School of Physics, Universiti Sains Malaysia, 11800 USM, Penang, Malaysia

## Abstract

In the title compound, C_23_H_23_NO_5_S·0.125H_2_O, the pyrrolidine ring has a twist conformation and the dihydro­pyran ring adopts a half-chair conformation; the two rings are *cis*-fused. The mol­ecule adopts a folded conformation. The sulfonyl-bound phenyl ring and the pyran ring of the coumarin ring system are stacked over one another, with a centroid–centroid distance of 3.7470 (7) Å; the dihedral angle between the two rings is 18.93 (2)°. An intra­molecular C—H⋯O hydrogen bond is observed. The solvent water mol­ecule, lying on a twofold rotation axis, is only partially occupied with an occupancy of 0.125 (relative occupancy with respect to the main molecule) and is involved in O—H⋯O and C—H⋯O hydrogen bonding.

## Related literature

For the biological activity of pyran­ocoumarin compounds, see: Kawaii *et al.* (2001 ▶); Hossain *et al.* (1996 ▶); Goel *et al.* (1997 ▶); Su *et al.* (2009 ▶); Xu *et al.* (2006 ▶). For asymmetry parameters, see: Duax *et al.* (1976 ▶).
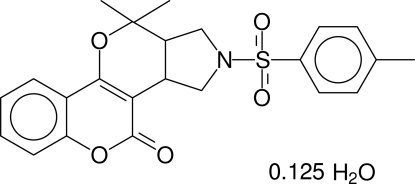


## Experimental

### 

#### Crystal data

C_23_H_23_NO_5_S·0.125H_2_O
*M* *_r_* = 427.74
Tetragonal, 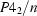

*a* = 15.1932 (2) Å
*c* = 17.7180 (3) Å
*V* = 4089.91 (10) Å^3^
*Z* = 8
Mo *K*α radiation
μ = 0.20 mm^−1^
*T* = 100 K
0.50 × 0.44 × 0.13 mm

#### Data collection

Bruker SMART APEXII CCD area-detector diffractometer
Absorption correction: multi-scan (*SADABS*; Bruker, 2005 ▶) *T*_min_ = 0.845, *T*_max_ = 0.976
97892 measured reflections
6004 independent reflections
5114 reflections with *I* > 2σ(*I*)
*R*_int_ = 0.058

#### Refinement

*R*[*F* ^2^ > 2σ(*F* ^2^)] = 0.039
*wR*(*F* ^2^) = 0.109
*S* = 1.05
6004 reflections
282 parameters
1 restraint
H atoms treated by a mixture of independent and constrained refinement
Δρ_max_ = 0.44 e Å^−3^
Δρ_min_ = −0.36 e Å^−3^

### 

Data collection: *APEX2* (Bruker, 2005 ▶); cell refinement: *SAINT* (Bruker, 2005 ▶); data reduction: *SAINT*; program(s) used to solve structure: *SHELXTL* (Sheldrick, 2008 ▶); program(s) used to refine structure: *SHELXTL*; molecular graphics: *SHELXTL*; software used to prepare material for publication: *SHELXTL* and *PLATON* (Spek, 2009 ▶).

## Supplementary Material

Crystal structure: contains datablocks global, I. DOI: 10.1107/S1600536809044730/bt5116sup1.cif

Structure factors: contains datablocks I. DOI: 10.1107/S1600536809044730/bt5116Isup2.hkl

Additional supplementary materials:  crystallographic information; 3D view; checkCIF report

## Figures and Tables

**Table 1:** Hydrogen-bond geometry (Å, °)

*D*—H⋯*A*	*D*—H	H⋯*A*	*D*⋯*A*	*D*—H⋯*A*
C4—H4*A*⋯O5	0.97	2.47	3.0588 (15)	119
O1*W*—H1*W*1⋯O2	0.83 (2)	2.05 (8)	2.837 (2)	161 (8)
C16—H16*B*⋯O1*W*^i^	0.96	2.43	3.345 (5)	160
C16—H16*B*⋯O1*W*^ii^	0.96	2.43	3.345 (5)	160
C14—H14*B*⋯O1*W*^iii^	0.96	2.44	3.282 (2)	147
C14—H14*B*⋯O1*W*^iv^	0.96	2.44	3.282 (2)	147

Symmetry codes: (i) 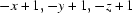; (ii) 
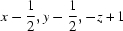
; (iii) 
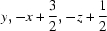
; (iv) 
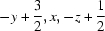
.

**Fig. 1.**
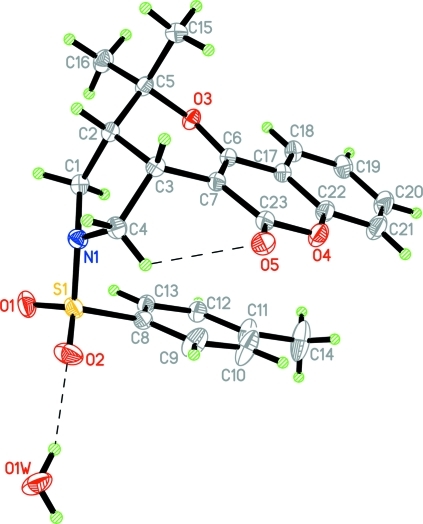
The molecular structure of the title compound. Displacement ellipsoids are drawn at the 50% probability level. The water molecule has a fractional occupancy of 0.125. One of the water H atoms is generated by the symmetry operation (3/2 - x, 3/2 - y, z). Dashed lines indicate hydrogen bonds.

**Fig. 2.**
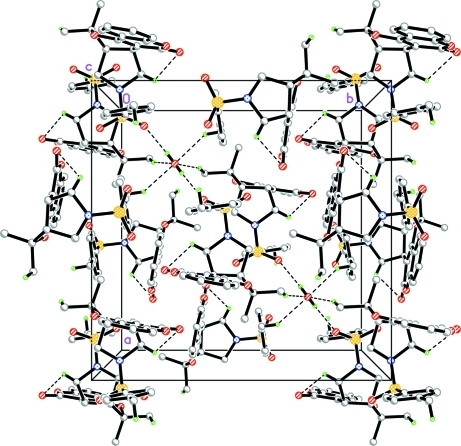
Crystal packing of the title compound, viewed along the c axis. The water molecule has a fractional occupancy of 0.125. Hydrogen bonds are shown as dashed lines. For the sake of clarity, H atoms not involved in the interactions have been omitted.

## supplementary crystallographic information

### Comment

Pyranocoumarins show strong activity against cancer cell lines (Kawaii *et al.*, 2001) and exhibit monoamine oxidase inhibitory activity (Hossain *et al.*, 1996). Antiulcer activity of some naturally occurring pyran*o*-coumarin has been reported (Goel *et al.*, 1997). They also show anti-hepatitis B virus and cytotoxic activities (Su *et al.*, 2009) and anti-TB activity (Xu *et al.*, 2006). We report here the crystal structure of the title pyranocoumarin derivative.

In the title molecule (Fig.1), the coumarin ring system is planar with an r.m.s. deviation of 0.036 Å. The pyrrolidine ring has a twist conformation, with asymmetry parameter (Duax *et al.*, 1976) ΔC_2_[N1] = ΔC_2_[C2—C3] = 1.7 (1)°. The tosyl group is equatorially attached to the pyrrolidine ring. The dihydropyran ring adopts a half-chair conformation, with the asymmetry parameter ΔC_2_[C2—C5] = 7.4 (1)°. The pyrrolidine ring is *cis*-fused to the dihydropyran ring. The sulfonyl group has a distorted tetrahedral geometry [O1—S1—O2 = 119.79 (6)°]. The molecule adopts a folded conformation, with the sulfonyl-bound phenyl ring and the pyran ring of the coumarin ring system being stacked over one another. The dihedral angle between the above two rings is 18.93 (2)° and their centroid-to-centroid separation is 3.7470 (7) Å. An intramolecular C4—H4A···O5 hydrogen bond is observed.

In the crystal structure, the water molecule with a fractional occupancy of 0.125 is involved in O—H···O and C—H···O hydrogen bonding with the coumarin derivative leading to the formation of a three-dimensional network (Fig.2).

### Experimental

To a solution of 4-hydroxycoumarin (1 mmol) in toluene (20 ml), the corresponding 2-(*N*-prenyl-*N*-tosylamino)acetaldehyde (1 mmol) and a catalytic amount of the base ethylenediamine-*N*,*N*'-diacetate (EDDA, 1 mmol) were added and the reaction mixture was refluxed for 12 h. After completion of the reaction, the solvent was evaporated under reduced pressure and the crude product was chromatographed using a hexane-ethyl acetate (8:2 *v*/*v*) mixture to obtain the title compound. The compound was recrystallized from ethyl acetate solution by slow evaporation.

### Refinement

The water H atom was located in a difference map and its positional parameters were refined with a O—H distance restraint of 0.84 (2) Å. The water molecule has a fractional occupancy of 1/8, which was initially refined and later fixed. The *U*_iso_ values were set equal to 1.5*U*_eq_ of the carrier atom for methyl and water H atoms and 1.2*U*_eq_ for the remaining H atoms. A rotating group model was used for the methyl groups.

### Crystal data

**Table:**

C_23_H_23_NO_5_S·0.125H_2_O	*D*_x_ = 1.389 Mg m^−^^3^
*M**_r_* = 427.74	Mo *K*α radiation, λ = 0.71073 Å
Tetragonal, *P*4_2_/*n*	Cell parameters from 9971 reflections
Hall symbol: -P 4bc	θ = 2.2–29.7°
*a* = 15.1932 (2) Å	µ = 0.20 mm^−^^1^
*c* = 17.7180 (3) Å	*T* = 100 K
*V* = 4089.91 (10) Å^3^	Block, colourless
*Z* = 8	0.50 × 0.44 × 0.13 mm
*F*(000) = 1802	

### Data collection

**Table:**

Bruker SMART APEXII CCD area-detector diffractometer	6004 independent reflections
Radiation source: fine-focus sealed tube	5114 reflections with *I* > 2σ(*I*)
graphite	*R*_int_ = 0.058
φ and ω scans	θ_max_ = 30.1°, θ_min_ = 1.8°
Absorption correction: multi-scan (*SADABS*; Bruker, 2005)	*h* = −19→21
*T*_min_ = 0.845, *T*_max_ = 0.976	*k* = −19→21
97892 measured reflections	*l* = −24→25

### Refinement

**Table:**

Refinement on *F*^2^	Primary atom site location: structure-invariant direct methods
Least-squares matrix: full	Secondary atom site location: difference Fourier map
*R*[*F*^2^ > 2σ(*F*^2^)] = 0.039	Hydrogen site location: inferred from neighbouring sites
*wR*(*F*^2^) = 0.109	H atoms treated by a mixture of independent and constrained refinement
*S* = 1.05	*w* = 1/[σ^2^(*F*_o_^2^) + (0.0592*P*)^2^ + 1.3394*P*] where *P* = (*F*_o_^2^ + 2*F*_c_^2^)/3
6004 reflections	(Δ/σ)_max_ = 0.001
282 parameters	Δρ_max_ = 0.44 e Å^−^^3^
1 restraint	Δρ_min_ = −0.36 e Å^−^^3^

### Special details

**Table:**

Geometry. All e.s.d.'s (except the e.s.d. in the dihedral angle between two l.s. planes) are estimated using the full covariance matrix. The cell e.s.d.'s are taken into account individually in the estimation of e.s.d.'s in distances, angles and torsion angles; correlations between e.s.d.'s in cell parameters are only used when they are defined by crystal symmetry. An approximate (isotropic) treatment of cell e.s.d.'s is used for estimating e.s.d.'s involving l.s. planes.
Refinement. Refinement of *F*^2^ against ALL reflections. The weighted *R*-factor *wR* and goodness of fit *S* are based on *F*^2^, conventional *R*-factors *R* are based on *F*, with *F* set to zero for negative *F*^2^. The threshold expression of *F*^2^ > σ(*F*^2^) is used only for calculating *R*-factors(gt) *etc*. and is not relevant to the choice of reflections for refinement. *R*-factors based on *F*^2^ are statistically about twice as large as those based on *F*, and *R*- factors based on ALL data will be even larger.

### Fractional atomic coordinates and isotropic or equivalent isotropic displacement parameters (Å^2^)

**Table:**

	*x*	*y*	*z*	*U*_iso_*/*U*_eq_	Occ. (<1)
S1	0.581635 (19)	0.56278 (2)	0.395952 (15)	0.02140 (8)	
O1	0.62409 (6)	0.47960 (7)	0.40829 (5)	0.0292 (2)	
O2	0.61326 (6)	0.63995 (7)	0.43408 (5)	0.0313 (2)	
O3	0.30418 (6)	0.47680 (5)	0.26574 (4)	0.01818 (16)	
O4	0.35320 (6)	0.73448 (6)	0.20657 (5)	0.02448 (19)	
O5	0.35668 (6)	0.77642 (6)	0.32563 (5)	0.02305 (18)	
N1	0.47990 (6)	0.55091 (7)	0.42058 (5)	0.01870 (19)	
C1	0.43048 (8)	0.47357 (8)	0.39346 (6)	0.0190 (2)	
H1A	0.4416	0.4226	0.4250	0.023*	
H1B	0.4460	0.4595	0.3418	0.023*	
C2	0.33383 (7)	0.50282 (7)	0.39921 (6)	0.0168 (2)	
H2	0.3125	0.4905	0.4503	0.020*	
C3	0.33722 (7)	0.60314 (7)	0.38784 (6)	0.0165 (2)	
H3	0.2865	0.6312	0.4120	0.020*	
C4	0.42234 (8)	0.62880 (8)	0.42864 (6)	0.0191 (2)	
H4A	0.4489	0.6801	0.4054	0.023*	
H4B	0.4111	0.6414	0.4814	0.023*	
C5	0.27372 (8)	0.45698 (7)	0.34279 (6)	0.0180 (2)	
C6	0.32304 (7)	0.56163 (7)	0.25100 (6)	0.0161 (2)	
C7	0.33841 (7)	0.62372 (7)	0.30463 (6)	0.0163 (2)	
C8	0.58331 (7)	0.58347 (8)	0.29825 (6)	0.0191 (2)	
C9	0.56995 (11)	0.66848 (9)	0.27189 (7)	0.0313 (3)	
H9	0.5634	0.7150	0.3056	0.038*	
C10	0.56654 (12)	0.68315 (9)	0.19508 (8)	0.0387 (4)	
H10	0.5564	0.7399	0.1775	0.046*	
C11	0.57777 (10)	0.61547 (9)	0.14336 (7)	0.0284 (3)	
C12	0.59130 (8)	0.53099 (8)	0.17059 (7)	0.0211 (2)	
H12	0.5989	0.4848	0.1367	0.025*	
C13	0.59373 (8)	0.51415 (8)	0.24766 (6)	0.0195 (2)	
H13	0.6022	0.4571	0.2653	0.023*	
C14	0.57659 (14)	0.63433 (12)	0.05996 (8)	0.0496 (5)	
H14A	0.5511	0.5853	0.0337	0.074*	
H14B	0.6357	0.6435	0.0425	0.074*	
H14C	0.5422	0.6862	0.0505	0.074*	
C15	0.17906 (8)	0.48882 (8)	0.34908 (7)	0.0229 (2)	
H15A	0.1432	0.4582	0.3130	0.034*	
H15B	0.1768	0.5509	0.3391	0.034*	
H15C	0.1575	0.4775	0.3991	0.034*	
C16	0.27884 (9)	0.35747 (8)	0.34841 (7)	0.0244 (2)	
H16A	0.2400	0.3315	0.3119	0.037*	
H16B	0.2617	0.3393	0.3982	0.037*	
H16C	0.3381	0.3386	0.3387	0.037*	
C17	0.32585 (7)	0.58344 (8)	0.17165 (6)	0.0180 (2)	
C18	0.31166 (8)	0.52190 (8)	0.11395 (6)	0.0211 (2)	
H18	0.3007	0.4633	0.1258	0.025*	
C19	0.31403 (9)	0.54850 (9)	0.03950 (7)	0.0252 (3)	
H19	0.3041	0.5078	0.0013	0.030*	
C20	0.33121 (10)	0.63606 (10)	0.02134 (7)	0.0307 (3)	
H20	0.3327	0.6532	−0.0290	0.037*	
C21	0.34609 (10)	0.69774 (9)	0.07730 (7)	0.0307 (3)	
H21	0.3584	0.7560	0.0651	0.037*	
C22	0.34229 (8)	0.67083 (8)	0.15238 (6)	0.0223 (2)	
C23	0.35020 (8)	0.71478 (8)	0.28241 (6)	0.0190 (2)	
O1W	0.7500	0.7500	0.4887 (3)	0.0379 (14)	0.25
H1W1	0.714 (5)	0.723 (5)	0.463 (4)	0.057*	0.25

### Atomic displacement parameters (Å^2^)

**Table:**

	*U*^11^	*U*^22^	*U*^33^	*U*^12^	*U*^13^	*U*^23^
S1	0.01678 (14)	0.03109 (17)	0.01633 (13)	0.00006 (10)	−0.00130 (9)	0.00123 (10)
O1	0.0219 (4)	0.0431 (6)	0.0225 (4)	0.0104 (4)	−0.0003 (3)	0.0089 (4)
O2	0.0238 (5)	0.0462 (6)	0.0241 (4)	−0.0101 (4)	−0.0015 (3)	−0.0076 (4)
O3	0.0253 (4)	0.0136 (4)	0.0156 (3)	−0.0018 (3)	0.0003 (3)	0.0007 (3)
O4	0.0349 (5)	0.0179 (4)	0.0207 (4)	−0.0063 (3)	−0.0050 (3)	0.0041 (3)
O5	0.0261 (4)	0.0163 (4)	0.0267 (4)	−0.0019 (3)	−0.0023 (3)	−0.0015 (3)
N1	0.0163 (4)	0.0216 (5)	0.0182 (4)	0.0010 (4)	−0.0001 (3)	0.0003 (3)
C1	0.0199 (5)	0.0190 (5)	0.0181 (5)	0.0011 (4)	−0.0008 (4)	0.0009 (4)
C2	0.0188 (5)	0.0164 (5)	0.0152 (4)	0.0000 (4)	0.0006 (4)	0.0013 (4)
C3	0.0178 (5)	0.0157 (5)	0.0161 (4)	0.0005 (4)	0.0008 (4)	−0.0007 (4)
C4	0.0208 (5)	0.0195 (5)	0.0169 (5)	0.0003 (4)	−0.0013 (4)	−0.0022 (4)
C5	0.0214 (5)	0.0162 (5)	0.0164 (5)	−0.0020 (4)	0.0005 (4)	0.0027 (4)
C6	0.0161 (5)	0.0148 (5)	0.0173 (5)	0.0006 (4)	−0.0008 (4)	0.0018 (4)
C7	0.0167 (5)	0.0152 (5)	0.0171 (5)	0.0003 (4)	−0.0011 (4)	0.0013 (4)
C8	0.0175 (5)	0.0223 (5)	0.0175 (5)	−0.0005 (4)	0.0009 (4)	0.0016 (4)
C9	0.0501 (9)	0.0184 (6)	0.0253 (6)	0.0008 (5)	0.0106 (6)	−0.0021 (5)
C10	0.0695 (11)	0.0195 (6)	0.0272 (6)	0.0099 (6)	0.0134 (7)	0.0065 (5)
C11	0.0404 (8)	0.0249 (6)	0.0200 (5)	0.0089 (5)	0.0051 (5)	0.0053 (5)
C12	0.0240 (6)	0.0204 (5)	0.0190 (5)	0.0016 (4)	0.0005 (4)	−0.0004 (4)
C13	0.0201 (5)	0.0183 (5)	0.0201 (5)	0.0014 (4)	0.0004 (4)	0.0027 (4)
C14	0.0861 (14)	0.0409 (9)	0.0217 (6)	0.0300 (9)	0.0102 (7)	0.0097 (6)
C15	0.0194 (5)	0.0235 (6)	0.0256 (6)	−0.0029 (4)	−0.0004 (4)	0.0019 (4)
C16	0.0322 (7)	0.0161 (5)	0.0250 (6)	−0.0025 (4)	−0.0019 (5)	0.0029 (4)
C17	0.0184 (5)	0.0194 (5)	0.0162 (5)	−0.0007 (4)	−0.0012 (4)	0.0021 (4)
C18	0.0237 (6)	0.0209 (5)	0.0188 (5)	0.0017 (4)	−0.0024 (4)	0.0008 (4)
C19	0.0290 (6)	0.0290 (6)	0.0177 (5)	0.0018 (5)	−0.0036 (4)	−0.0004 (4)
C20	0.0389 (8)	0.0351 (7)	0.0180 (5)	−0.0040 (6)	−0.0036 (5)	0.0072 (5)
C21	0.0424 (8)	0.0273 (7)	0.0225 (6)	−0.0088 (5)	−0.0046 (5)	0.0090 (5)
C22	0.0266 (6)	0.0207 (6)	0.0195 (5)	−0.0039 (4)	−0.0039 (4)	0.0030 (4)
C23	0.0188 (5)	0.0173 (5)	0.0208 (5)	−0.0010 (4)	−0.0023 (4)	0.0027 (4)
O1W	0.055 (4)	0.037 (3)	0.022 (2)	−0.029 (3)	0.000	0.000

### Geometric parameters (Å, °)

**Table:**

S1—O1	1.4355 (10)	C9—C10	1.3799 (19)
S1—O2	1.4359 (10)	C9—H9	0.93
S1—N1	1.6161 (10)	C10—C11	1.3878 (19)
S1—C8	1.7597 (12)	C10—H10	0.93
O3—C6	1.3459 (13)	C11—C12	1.3865 (17)
O3—C5	1.4727 (13)	C11—C14	1.5054 (18)
O4—C22	1.3728 (14)	C12—C13	1.3897 (15)
O4—C23	1.3774 (14)	C12—H12	0.93
O5—C23	1.2137 (14)	C13—H13	0.93
N1—C1	1.4749 (15)	C14—H14A	0.96
N1—C4	1.4784 (15)	C14—H14B	0.96
C1—C2	1.5376 (16)	C14—H14C	0.96
C1—H1A	0.97	C15—H15A	0.96
C1—H1B	0.97	C15—H15B	0.96
C2—C5	1.5225 (15)	C15—H15C	0.96
C2—C3	1.5382 (15)	C16—H16A	0.96
C2—H2	0.98	C16—H16B	0.96
C3—C7	1.5072 (14)	C16—H16C	0.96
C3—C4	1.5320 (16)	C17—C22	1.3935 (16)
C3—H3	0.98	C17—C18	1.4021 (16)
C4—H4A	0.97	C18—C19	1.3800 (16)
C4—H4B	0.97	C18—H18	0.93
C5—C16	1.5172 (16)	C19—C20	1.3934 (19)
C5—C15	1.5215 (17)	C19—H19	0.93
C6—C7	1.3592 (15)	C20—C21	1.383 (2)
C6—C17	1.4449 (14)	C20—H20	0.93
C7—C23	1.4497 (15)	C21—C22	1.3929 (16)
C8—C9	1.3883 (17)	C21—H21	0.93
C8—C13	1.3920 (16)	O1W—H1W1	0.83 (2)
			
O1—S1—O2	119.79 (6)	C8—C9—H9	120.4
O1—S1—N1	106.89 (6)	C9—C10—C11	121.80 (12)
O2—S1—N1	106.50 (6)	C9—C10—H10	119.1
O1—S1—C8	107.49 (6)	C11—C10—H10	119.1
O2—S1—C8	108.18 (6)	C12—C11—C10	118.31 (12)
N1—S1—C8	107.42 (5)	C12—C11—C14	121.30 (12)
C6—O3—C5	116.27 (8)	C10—C11—C14	120.38 (12)
C22—O4—C23	121.68 (9)	C11—C12—C13	121.08 (11)
C1—N1—C4	111.59 (9)	C11—C12—H12	119.5
C1—N1—S1	119.20 (8)	C13—C12—H12	119.5
C4—N1—S1	120.17 (8)	C12—C13—C8	119.36 (11)
N1—C1—C2	103.55 (9)	C12—C13—H13	120.3
N1—C1—H1A	111.1	C8—C13—H13	120.3
C2—C1—H1A	111.1	C11—C14—H14A	109.5
N1—C1—H1B	111.1	C11—C14—H14B	109.5
C2—C1—H1B	111.1	H14A—C14—H14B	109.5
H1A—C1—H1B	109.0	C11—C14—H14C	109.5
C5—C2—C1	113.40 (9)	H14A—C14—H14C	109.5
C5—C2—C3	112.79 (9)	H14B—C14—H14C	109.5
C1—C2—C3	104.22 (9)	C5—C15—H15A	109.5
C5—C2—H2	108.7	C5—C15—H15B	109.5
C1—C2—H2	108.7	H15A—C15—H15B	109.5
C3—C2—H2	108.7	C5—C15—H15C	109.5
C7—C3—C4	113.50 (9)	H15A—C15—H15C	109.5
C7—C3—C2	109.51 (9)	H15B—C15—H15C	109.5
C4—C3—C2	102.63 (9)	C5—C16—H16A	109.5
C7—C3—H3	110.3	C5—C16—H16B	109.5
C4—C3—H3	110.3	H16A—C16—H16B	109.5
C2—C3—H3	110.3	C5—C16—H16C	109.5
N1—C4—C3	104.48 (9)	H16A—C16—H16C	109.5
N1—C4—H4A	110.9	H16B—C16—H16C	109.5
C3—C4—H4A	110.9	C22—C17—C18	118.96 (10)
N1—C4—H4B	110.9	C22—C17—C6	117.53 (10)
C3—C4—H4B	110.9	C18—C17—C6	123.50 (10)
H4A—C4—H4B	108.9	C19—C18—C17	119.85 (11)
O3—C5—C16	104.38 (9)	C19—C18—H18	120.1
O3—C5—C15	107.45 (9)	C17—C18—H18	120.1
C16—C5—C15	111.13 (10)	C18—C19—C20	120.34 (12)
O3—C5—C2	109.06 (9)	C18—C19—H19	119.8
C16—C5—C2	112.46 (9)	C20—C19—H19	119.8
C15—C5—C2	111.93 (9)	C21—C20—C19	120.80 (11)
O3—C6—C7	124.44 (10)	C21—C20—H20	119.6
O3—C6—C17	114.50 (9)	C19—C20—H20	119.6
C7—C6—C17	121.06 (10)	C20—C21—C22	118.62 (12)
C6—C7—C23	119.59 (10)	C20—C21—H21	120.7
C6—C7—C3	122.54 (10)	C22—C21—H21	120.7
C23—C7—C3	117.72 (9)	O4—C22—C21	117.14 (11)
C9—C8—C13	120.26 (11)	O4—C22—C17	121.43 (10)
C9—C8—S1	119.68 (9)	C21—C22—C17	121.41 (11)
C13—C8—S1	119.99 (9)	O5—C23—O4	116.44 (10)
C10—C9—C8	119.17 (12)	O5—C23—C7	125.11 (11)
C10—C9—H9	120.4	O4—C23—C7	118.45 (10)
			
O1—S1—N1—C1	−47.47 (10)	O2—S1—C8—C9	−31.08 (12)
O2—S1—N1—C1	−176.63 (8)	N1—S1—C8—C9	83.53 (12)
C8—S1—N1—C1	67.65 (10)	O1—S1—C8—C13	21.34 (11)
O1—S1—N1—C4	168.19 (8)	O2—S1—C8—C13	152.02 (10)
O2—S1—N1—C4	39.03 (10)	N1—S1—C8—C13	−93.37 (10)
C8—S1—N1—C4	−76.69 (9)	C13—C8—C9—C10	0.5 (2)
C4—N1—C1—C2	−10.44 (11)	S1—C8—C9—C10	−176.44 (12)
S1—N1—C1—C2	−157.62 (7)	C8—C9—C10—C11	−1.3 (3)
N1—C1—C2—C5	152.39 (9)	C9—C10—C11—C12	1.1 (3)
N1—C1—C2—C3	29.36 (10)	C9—C10—C11—C14	−178.00 (17)
C5—C2—C3—C7	−39.60 (12)	C10—C11—C12—C13	−0.1 (2)
C1—C2—C3—C7	83.82 (10)	C14—C11—C12—C13	178.96 (14)
C5—C2—C3—C4	−160.46 (9)	C11—C12—C13—C8	−0.66 (19)
C1—C2—C3—C4	−37.03 (10)	C9—C8—C13—C12	0.48 (18)
C1—N1—C4—C3	−12.70 (12)	S1—C8—C13—C12	177.37 (9)
S1—N1—C4—C3	134.11 (8)	O3—C6—C17—C22	178.02 (10)
C7—C3—C4—N1	−87.73 (11)	C7—C6—C17—C22	−1.85 (16)
C2—C3—C4—N1	30.35 (10)	O3—C6—C17—C18	−0.97 (16)
C6—O3—C5—C16	−166.93 (9)	C7—C6—C17—C18	179.16 (11)
C6—O3—C5—C15	74.98 (11)	C22—C17—C18—C19	−0.09 (18)
C6—O3—C5—C2	−46.55 (12)	C6—C17—C18—C19	178.89 (11)
C1—C2—C5—O3	−60.12 (12)	C17—C18—C19—C20	0.55 (19)
C3—C2—C5—O3	58.06 (12)	C18—C19—C20—C21	0.0 (2)
C1—C2—C5—C16	55.17 (12)	C19—C20—C21—C22	−0.9 (2)
C3—C2—C5—C16	173.35 (9)	C23—O4—C22—C21	179.72 (12)
C1—C2—C5—C15	−178.89 (9)	C23—O4—C22—C17	1.34 (18)
C3—C2—C5—C15	−60.71 (12)	C20—C21—C22—O4	−176.96 (13)
C5—O3—C6—C7	18.19 (15)	C20—C21—C22—C17	1.4 (2)
C5—O3—C6—C17	−161.67 (9)	C18—C17—C22—O4	177.39 (11)
O3—C6—C7—C23	−174.40 (10)	C6—C17—C22—O4	−1.65 (17)
C17—C6—C7—C23	5.45 (16)	C18—C17—C22—C21	−0.91 (19)
O3—C6—C7—C3	0.98 (17)	C6—C17—C22—C21	−179.95 (12)
C17—C6—C7—C3	−179.17 (10)	C22—O4—C23—O5	−176.80 (11)
C4—C3—C7—C6	124.43 (11)	C22—O4—C23—C7	2.28 (16)
C2—C3—C7—C6	10.40 (15)	C6—C7—C23—O5	173.33 (11)
C4—C3—C7—C23	−60.11 (13)	C3—C7—C23—O5	−2.27 (17)
C2—C3—C7—C23	−174.13 (10)	C6—C7—C23—O4	−5.67 (16)
O1—S1—C8—C9	−161.76 (11)	C3—C7—C23—O4	178.73 (10)

### Hydrogen-bond geometry (Å, °)

**Table:**

*D*—H···*A*	*D*—H	H···*A*	*D*···*A*	*D*—H···*A*
C4—H4A···O5	0.97	2.47	3.0588 (15)	119
O1W—H1W1···O2	0.83 (2)	2.05 (8)	2.837 (2)	161 (8)
C16—H16B···O1W^i^	0.96	2.43	3.345 (5)	160
C16—H16B···O1W^ii^	0.96	2.43	3.345 (5)	160
C14—H14B···O1W^iii^	0.96	2.44	3.282 (2)	147
C14—H14B···O1W^iv^	0.96	2.44	3.282 (2)	147

Symmetry codes: (i) −*x*+1, −*y*+1, −*z*+1; (ii) *x*−1/2, *y*−1/2, −*z*+1; (iii) *y*, −*x*+3/2, −*z*+1/2; (iv) −*y*+3/2, *x*, −*z*+1/2.
